# Migration tracking reveals geographic variation in the vulnerability of a Nearctic-Neotropical migrant bird

**DOI:** 10.1038/s41598-020-62132-6

**Published:** 2020-03-26

**Authors:** Diana L. Humple, Renée L. Cormier, T. Will Richardson, Ryan D. Burnett, Nathaniel E. Seavy, Kristen E. Dybala, Thomas Gardali

**Affiliations:** 1 Point Blue Conservation Science, 3820 Cypress Drive # 11, Petaluma, CA 94954 USA; 2 Tahoe Institute for Natural Science, 948 Incline Way, Incline Village, NV 89451 USA; 3 Present Address: National Audubon Society, 220 Montgomery St, Suite 1000, San Francisco, CA 94104 USA

**Keywords:** Animal migration, Conservation biology

## Abstract

We compared the vulnerability of a Nearctic-Neotropical migrant (Swainson’s Thrush, *Catharus ustulatus*) for three geographically-defined breeding populations in California by linking breeding and wintering regions, estimating migration distances, and quantifying relative forest loss. Using data from light-level geolocator and GPS tags, we found that breeding birds from the relatively robust coastal population in the San Francisco Bay area wintered predominantly in western Mexico (n = 18), whereas the far rarer breeding birds from two inland populations that occur near one another in the Sierra Nevada and southern Cascades mountain ranges migrated to farther wintering destinations, with birds from the Lassen region (n = 5) predominantly going to Central America and birds from the Tahoe region (n = 7) predominantly to South America. Landscape-level relative forest loss was greater in the breeding and wintering regions of the two Cascade-Sierra populations than those of coastal birds. Longer migration distances and greater exposure to recent forest loss suggest greater current vulnerability of Cascade-Sierra birds. Our results demonstrate that for some species, quantifying migration distances and destinations across relatively small distances among breeding populations (in this case, 140–250 km apart) can identify dramatically different vulnerabilities that need to be considered in conservation planning.

## Introduction

Quantifying the vulnerability of wildlife populations to environmental change is one step toward identifying and prioritizing management actions designed to preserve biodiversity^1–3^. Typically, vulnerability assessments synthesize information on exposure (extrinsic factors), sensitivity (intrinsic traits), and adaptive capacity (evolutionary potential or plasticity) of species or populations^4^. For migratory species, such information also must relate to geographies and movement ecology covering their full life cycle^5,6^.

For many small migratory birds, our understanding of vulnerability has been limited by the paucity of information available on migratory connectivity between breeding and non-breeding regions for geographically-defined populations. For decades, there has been speculation that non-breeding habitat loss may explain widespread Nearctic-Neotropical-migrant declines^7^, while other studies point to limitations on the breeding grounds^8,9^. Over the past decade, the miniaturization of tracking technology has provided novel opportunities to test such hypotheses by linking declines in one region to habitat change in another region during a different portion of their annual cycle^10,11^.

We illustrate an approach to using tracking data to investigate spatial variation in vulnerability to environmental change, with a Nearctic-Neotropical migrant species that varies considerably in abundance and trends across its breeding range. The Swainson’s Thrush (*Catharus ustulatus*) breeds primarily in densely vegetated riparian habitat across parts of North America, and winters from Mexico to South America^12^. Variation in population trends suggest that Swainson’s Thrush populations are less robust and more vulnerable in some regions of California than others. On the central coast, they are common and the population relatively stable^13–15^; whereas in the Sierra Nevada and contiguous southern Cascades mountain ranges (hereafter the Cascade-Sierra), they are patchily distributed, rare, and found in low densities where they occur, even where presumably appropriate habitat exists^16^. Historic accounts suggest the Swainson’s Thrush was once a much more common breeder there (at least in the Sierra Nevada where there are more accounts)^17–19^ and has undergone local extirpations and significant declines that span decades^20,21^. The loss and degradation of riparian habitat on the breeding grounds in California^12^, as well as deforestation of wintering habitat of populations breeding in the Cascade-Sierra^16,21^, have been hypothesized as drivers of Swainson’s Thrush extirpations and declines.

Here, we assess spatial variation in vulnerability of Swainson’s Thrush breeding in northern California, using data from an earlier tracking study of coastal birds^22^ combined with additional tracking of coastal birds from the San Francisco Bay area, and novel tracking of two populations from the Tahoe and Lassen regions within the Cascade-Sierra. We then use what we learn about wintering locations to identify sensitivities based on conservative estimates of migration distance (assuming longer-distance migrants are more sensitive^2–4,6^) and exposure to relative landscape-level forest loss on both breeding and wintering grounds. Based on the differences in population size, distribution, and trends in these breeding regions, including from the authors’ own extensive observations across all three regions, we predicted different wintering locations for these populations, and that the two Cascade-Sierra populations would have greater sensitivity and exposure than coastal birds, and hence be more vulnerable.

## Methods

### Study location and field methods

**Figure 1:**
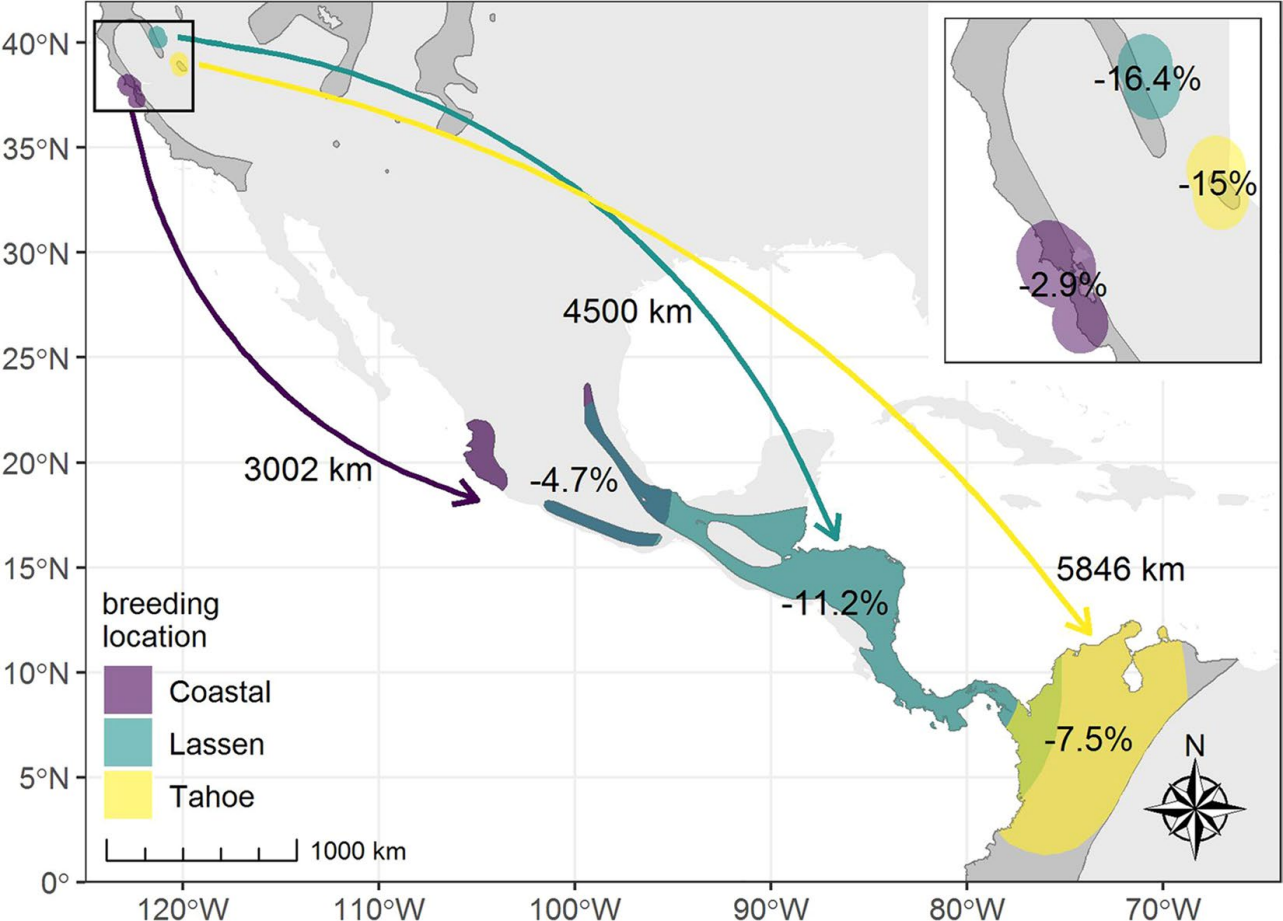
Estimated wintering destinations, relative forest loss (from 2000–2017)^32^, and migration distances for three breeding populations of Swainson’s Thrush in California. Dark gray polygon (often not visible beneath colored areas) indicates estimated breeding and winter ranges^31^; note, the authors know of additional breeding sites slightly beyond these boundaries, including where Lassen birds were tagged (see Supplementary Fig. S1); and the open polygon in middle of the Lassen wintering range is considered outside the known wintering range of the species. Dark teal reflects the area of potential overlap in wintering ranges of coastal and Lassen birds, and yellowish-green the area of potential overlap between Lassen and Tahoe birds. Exact breeding tag-deployment locations are buffered to quantify regional forest loss at a more appropriate scale, and wintering destinations reflect 95% kernel densities to account for estimations involved in light-level technology. Arrows connect corresponding breeding and wintering areas, and distances shown between breeding and wintering areas, represent great-circle distances between the centroids of each polygon; neither are intended to imply migratory routes.

We attached either light-level geolocator or Global Positioning System (GPS) tags to adult Swainson’s Thrushes in breeding condition in coastal California, and in the Tahoe and Lassen regions within California’s northern Sierra Nevada and southern Cascade Mountains (hereafter the Cascade-Sierra), in the summers of 2014–2015 (Fig. 1; Supplementary Table S1). Light-level tags collected light-intensity data from which latitude and longitude were coarsely estimated, and GPS tags were programmed to collect 8 positions from satellites, spanning the winter period. In the coastal region, we deployed tags in the San Francisco Bay area: 10 light-level tags south of San Francisco Bay in coastal San Mateo County in 2014, and 30 GPS tags north of the bay in the Point Reyes area of Marin County in 2015. This was the second phase in this effort in the Point Reyes area: light-level tags were previously deployed in 2010^22^, and those data are included in the results presented here. In the Cascade-Sierra, a mix of light-level and GPS tags were deployed in each of two regions: 11 light-level and 10 GPS tags in the Lassen region in the northern Sierra Nevada/southern Cascade Mountains, Plumas County, in 2015; and 24 light-level and 5 GPS tags farther south in the Tahoe region of the northern Sierra Nevada, Placer and El Dorado counties, in 2014 and 2015. On the coast, tag deployment and recovery was primarily done during normal operations at constant-effort mist-netting stations; and in the Cascade-Sierra, tag deployment and recovery was done via target netting, using song playback and decoys. Due to the differences in capture methods among the regions, sex ratios on the coast were fairly equal, whereas they were strongly male-biased in the Cascade-Sierra. See Supplementary Table S1 for more tagging details by location. The habitat at capture sites on the coast included riparian forest and Douglas-fir (*Pseudotsuga menziesii*) forest mixed with coastal scrub^23,24^, and in the Cascade-Sierra was dominated by dense riparian vegetation and wet riparian mountain meadows.

Light-level tags (developed by British Antarctic Survey or Migrate Technology Ltd) and PinPoint8 GPS tags (developed by Lotek Wireless) were attached with a leg-loop harness^25^ of StretchMagic jewelry cord of 1.0 mm gauge (light-level tags) or 0.7 mm gauge (GPS tags), and each harness sealed with a small crimped jewelry bead and super glue. The average weight of the harness and GPS tag together was 1.0 g, averaging 3.4% of the bird’s weight (n = 37); the average weight of the harness and light-level tag together was 0.8 g, averaging 2.8% of the bird’s weight (n = 18). Each bird was banded with a federal U.S. Geological Survey aluminum band; additionally, those in the coast and Lassen regions were also given either a unique or cohort color band, and those in the Tahoe region a unique combination of three color bands and one federal band. We determined age of each bird (only adults were tagged), determined sex using the presence of a brood patch (female) or cloacal protuberance (male), and weighed each bird to the nearest 0.1 g before and after the geolocator tag was attached. The technology requires recapturing birds following a full migration cycle, upon which we removed the tags, extracted the data, and collected the same information (age, sex, weight) as during the initial capture. Recovery occurred the following two spring/summer seasons after deployment. Capture and handling followed strict bird safety protocols in accordance with the North American Banding Council^26^. All banding and tagging was approved by the United States Geological Survey’s Bird Banding Laboratory (USGS BBL Permit Numbers: 09316 and 23272).

### Analysis

#### Daily location estimates (GPS tags)

We programmed the GPS tags to attempt to collect 8 GPS coordinates during the wintering period between 25 October and 25 March (date range based on previously-determined wintering arrival and departure dates^22^), to attempt to determine their wintering locations and potential within-winter movements^22,27^. We downloaded location estimates from recovered GPS tags using Lotek Wireless PinPoint Host software, revision 3 (2014). For tags with multiple (2–6) points, we used the mean latitude and longitude estimates; the maximum distance between points for a given individual was 2.8 km.

#### Daily location estimates (Light-level tags)

We used IntigeoIF software version 1.5.2 (Migrate Technology) to download the light intensity data. To analyze the data, we log-transformed the light values, and used the TwGeos package^28,29^ to identify twilight events. We then analyzed the light-level data with a Bayesian framework using the Solar/Satellite Geolocation for Animal Tracking (SGAT) package^30^; SGAT uses the twilight times calculated using the threshold method, the observed difference between the known twilight times and those calculated, and a movement model.

To identify twilight periods, we used a threshold value of 0.65 for all individuals; we selected a threshold as low as possible, but above most of the noise in the nighttime light levels. We used the twilightEdit function to delete twilights (sunrise/sunset events) if they occurred 15 min before or after the previous or next day’s twilight value; we used a 4-day window (2 days before and after), and set stationary site variation to 20 minutes^28^.

We used two on-bird calibration periods for each tag: the first calibration period was from the day after tag attachment to 31 August of the tagging year, before the bird departed for fall migration. The second calibration period began the year after deployment on 5 June, after which all birds appeared to be back on the breeding grounds, and ended the day before the bird was recaptured. For two birds, the tag failed before spring migration, so only the first calibration period was used. We estimated the zenith angle for each tag; the zenith angle is defined as the angle of the sun relative to the earth’s 90° vertical axis, when the light intensity data from the geolocator crosses a specific threshold (e.g., set at 0.65 for our tags). Using the defined calibration periods above, we estimated median zenith angles for each tag (range 94.8° to 96.5°), and used each tag’s estimated zenith angle to plot estimated positions for the entire tag deployment period.

For the SGAT analysis we used a movement model with probable flight speeds defined by a gamma distribution (mean of 2.2 and a SD of 0.25). We also used a land mask which limited the bird’s location to land during stationary periods. To generate the posterior distribution, we used 3 independent chains, 6000 samples for burn-in and tuning, and then based our analyses on a final run of 1000 samples.

For each individual, we checked that location results were relatively consistent between the simple threshold location estimates and the subsequent modeled estimates. We also checked that location estimates were not sensitive to small changes in model assumptions. Through this process, we excluded one tag for which wintering areas varied from as far north as Cuba to as far south as Peru depending on minor variations in the analysis method.

### Mapping breeding and wintering regions

We estimated the wintering regions for each breeding population (coastal, Lassen, and Tahoe) by creating a 95% kernel density estimate around all known (GPS) and estimated (light-level geolocator) wintering locations for both tag types. Similarly, we created a 95% kernel density estimate around all the known breeding tag deployment locations and further buffered these small areas by 20 km, to better capture the surrounding landscape and quantify regional forest loss at a more appropriate scale.

### Estimating forest loss

We clipped all breeding and wintering polygons to the published species range^31^ to allow us to focus our consideration of habitat change to the area within the species range. We then estimated relative landscape-level forest loss in each of these polygons separately using 2000–2017 data available from Global Forest Change^32^. The data are divided into 10×10 degree tiles with a spatial resolution of 1 arc-second per pixel (equivalent to approximately 30 m at the equator). Separate tiles contain information about the baseline canopy cover in the year 2000 (“baseline”) and the year in which any forested pixel became unforested (“lossyear”). We converted baseline cover from the year 2000 into a binary (0 or 1) reflecting whether or not each pixel was forested, using a minimum threshold of 50% forest cover. For each pixel forested in 2000, we also converted “lossyear” data into a binary (0 or 1) reflecting whether or not each pixel became unforested by 2017. For all pixels within each Swainson’s Thrush wintering area polygon, we summarized the number of pixels where forest was originally present in 2000, and the proportion of those pixels where forest was lost by 2017. Forest growth data were also available, but only through 2012; we assumed that 12 years was a relatively short time frame within which an unforested pixel could become forested, and therefore we ignored forest gains in this analysis.

### Measuring migration distance

As a conservative index of migration distance, we measured great-circle distances between the centroids of each breeding area polygon and its corresponding wintering area polygon. We do not know the actual route taken nor exact distances flown by each individual bird.

## Results

Tag recovery efforts in the subsequent two summers resulted in data from 4 GPS and 2 light-level tags from the coast, 4 light-level and 1 GPS tag from Lassen, and 7 light-level tags from Tahoe (Supplementary Table S1). An additional 3 GPS tags at Lassen and 6 GPS tags on the coast were recovered, but did not contain retrievable data. To these, we added data from 12 recovered light-level tags from our previous study in the Point Reyes area on the coast^22^. Kernel densities indicated the wintering range of the coastal-California breeding population of Swainson’s Thrushes, from both north and south of San Francisco Bay, as western Mexico; the Lassen breeding population as Central America, from southern Mexico to western Panama (possibly into western Colombia); and the Tahoe breeding population as northwestern South America (possibly into eastern Panama; Fig. 1; also see Supplementary Figs S1 and S2).

From our conservative estimates of migration distance, coastal birds migrated the shortest distance, with approximately 3,000 km between the centroids of the breeding and wintering locations; Lassen birds migrated an intermediate distance (4500 km); and those from Tahoe migrated the longest distance, nearly twice that of coastal birds (5800 km; Fig. 1). Recent (years 2000–2017) forest loss occurred in the wintering regions of all three breeding populations, and was greater for the two Cascade-Sierra breeding populations (11.2% for Lassen birds; 7.5% for Tahoe birds) than the coastal-breeding population (4.7%). On the breeding grounds, relative forest loss was greater for both Cascade-Sierra regions than on the coast (16.4% and 15%, vs 2.9%; Fig. 1).

## Discussion

Our results demonstrate the utility of migration-tracking data to identify spatial variability in the vulnerability of migratory birds, even among geographically-proximate breeding locations. Specifically, we suggest that Swainson’s Thrushes breeding in the Cascade-Sierra are currently more vulnerable to environmental change than coastal breeding birds, because they migrate longer distances and are exposed to a greater degree of relative forest loss on both their breeding and wintering regions. Our study further revealed that birds from the nearby Lassen and Tahoe regions within the Cascade-Sierra, whose closest study sites were a mere 140 km apart, migrate to fairly distinct regions within Central America (Lassen birds) and South America (Tahoe birds), and should be considered as distinct populations with respect to conservation. Without this tracking study, we would not know that these nearby populations should be considered as such. Given our observation of this potential migratory divide^27^ between Lassen and Tahoe birds, combined with what is known about the different migratory destinations of Swainson’s Thrush subspecies groups (with the russet-backed, or *Ustulatus*, group of subspecies wintering in Mexico and Central America and the olive-backed, or *Swainsoni*, group wintering in South America^12^) as well as the occurrence of subspecies hybrid zones in parts of their range^33^, further study is warranted into the genetics, subspecies, and potential intergrade zone in the Cascade-Sierra. In addition, our study determined that the wintering destinations of Cascade-Sierra populations differed greatly from those of the relatively proximate (240–270 km away) coastal-breeding thrushes, which migrate to western Mexico. Prior to this study, the wintering destinations for Swainson’s Thrushes in the Sierra-Cascade and vulnerability for this species in California had not been previously described.

Our estimate of exposure to landscape-level forest loss is intended as an index of ecosystem change. We used an existing dataset of relative forest loss to quantify exposure to habitat change across both breeding and wintering regions, which does not necessarily reflect or relate to finer-scale changes in or requirements of Swainson’s Thrush habitat (e.g., riparian or specific tropical-forest types^34^). Our results point to the need to develop better habitat models for migratory birds across all regions of their full life cycle. Such models would be particularly useful for Swainson’s Thrush in the Cascade-Sierra – whose breeding-habitat availability and quality are known to have declined due to fire suppression, grazing, hydrologic degradation, and development^35^ – given that the exact impacts of these habitat declines on these patchily-distributed birds is poorly understood. We also do not know what role historical patterns of forest loss in Central and South America^36^ might have played in the decades-long Swainson’s Thrush decline in the Cascade-Sierra; the more recent forest losses analyzed here are intended to indicate current pressures placed on already vulnerable populations.

In conservation assessments, migration distance is often used as an intrinsic indicator of vulnerability, with species that migrate longer distances considered more sensitive to environmental change (e.g., vulnerable to more episodes of severe weather, or more encounters with habitat loss, along a longer route)^4,37^. Our results suggest that even across relatively short distances among breeding populations, there may be spatial variation in vulnerability due to differences in migration distance – such as the heightened vulnerability revealed for Cascade-Sierra thrushes, especially the Tahoe population, due to their migration to a more distant wintering region. Therefore, care must be taken to not extend assumptions about migratory connectivity or vulnerability to nearby populations when those characteristics are only known across a limited portion of a species’ range. That said, our migration distance estimates between breeding and wintering regions should be used cautiously, as we simply calculated distances between the centroids of breeding and wintering regions and could not evaluate the actual routes of individuals. Additionally, because migration behavior is genetically controlled^38,39^, this spatial variation suggests evidence for phenotypic and genetic diversity across regions that should be considered by conservation strategies designed to maintain the resilience of a species to environmental change. Finally, conservation strategies for Swainson’s Thrush and other species should consider the influence of migration distance and wintering region when evaluating vulnerability to different future climate scenarios. Specifically, migratory birds depend on timing their movements with conditions that facilitate survival and reproduction^40^, and birds from different wintering regions may migrate to or arrive on breeding grounds at different times^27^; therefore, migration distance and wintering latitude may influence their ability to track conditions and shift phenology^41,42^.

Our study reveals important differences among three breeding Swainson’s Thrush populations in northern California, including in wintering destinations, distances between wintering and breeding regions, and landscape-level forest loss across breeding and wintering regions. These results suggest that the remaining Swainson’s Thrushes in the Cascade-Sierra are more vulnerable than coastal populations both because they migrate longer distances (especially Tahoe birds) and are exposed to greater degrees of forest loss (especially Lassen birds). As with many migratory species, enhancing populations of Cascade-Sierra Swainson’s Thrushes will require management actions across more than one region within their full life cycle^43,44^. The three wintering areas we identified were large and overlapping; this may result both from the real dispersion of the birds, but also from the relatively coarse location estimates derived from light-level geolocation. Our success in recovering more accurate locations from the subset of GPS loggers that produced data suggests that in the future, as GPS tags are improved and capable of collecting more points, more precise information on migration pathways and destinations will be available. Future studies evaluating genetic differentiation across the Cascade-Sierra Swainson’s Thrush range may also provide insight into migratory connectivity and vulnerability. Meanwhile, for Swainson’s Thrush and other migratory species, future population or genetic studies will benefit from considering direct migratory connectivity tracking results such as from this study, and the application of those results with respect to vulnerability. Recent and future advances in our ability to describe migratory connectivity provide the missing link that allows us to better interpret regional variation in population trends. Combining this knowledge, which is gained from direct tracking studies, with landscape analyses at appropriate spatial and temporal scales will improve conservation outcomes for all migratory species by helping identify spatial variation in specific vulnerabilities.

## Supplementary information

Supplementary Information.

## Data Availability

Light-level and GPS tag data, and associated metadata, have been uploaded to Movebank (www.movebank.org); metadata are available, and data available upon request.

## References

[1] Pereira HM, Daily GC, Roughgarden J (2004). A framework for assessing the relative vulnerability of species to land‐use change. Ecol. Applications..

[2] Wilson K (2005). Measuring and incorporating vulnerability into conservation planning. Environ. Management..

[3] Glick P., Stein, B. A. & Edelson, N. A. *Scanning the Conservation Horizon: A Guide to Climate Change Vulnerability Assessment*. Washington, D.C.: National Wildlife Federation. 168 p. (2011).

[4] Gardali T, DiGaudio R, Seavy NE, Comrack L (2012). A climate change vulnerability assessment of California’s at-risk birds. PLoS ONE..

[5] Small-Lorenz SL, Culp LA, Ryder TB, Will TC, Marra PP (2013). A blind spot in climate change vulnerability assessments. Nature Climate Change..

[6] Culp LA, Cohen EM, Scarpignato AL, Thogmartin WE, Marra PP (2017). Full annual cycle climate change vulnerability assessment for migratory birds. Ecosphere..

[7] Robbins CS, Sauer JR, Greenberg RS, Droege S (1989). Population declines in North American birds that migrate to the Neotropics. Proc. of the Nat. Academy of Sci..

[8] Johnson MD, Geupel GR (1996). The importance of productivity to the dynamics of a Swainson’s Thrush population. Condor..

[9] Gardali T, Ballard G, Nur N, Geupel GR (2000). Demography of a declining population of Warbling Vireos in coastal California. Condor.

[10] Taylor CM, Stutchbury BJ (2016). Effects of breeding versus winter habitat loss and fragmentation on the population dynamics of a migratory songbird. Ecolog. Applications..

[11] Kramer GR (2018). Population trends in Vermivora warblers are linked to strong migratory connectivity. Proc. of the Nat. Academy of Sci..

[12] Mack, D. E. & Yong, W. Swainson’s Thrush (*Catharus ustulatus*), version 2.0. In The Birds of North America (A.F. Poole and F.B. Gill, Editors). Cornell Lab of Ornithology, Ithaca, NY, USA. 10.2173/bna.540 (2000).

[13] RHJV (Riparian Habitat Joint Venture). Version 1.0. The Riparian Bird Conservation Plan: A Strategy for Reversing the Decline of Riparian Associated Birds in California. California Partners in Flight. http://www.prbo.org/CalPIF/Riparian/Riparian.html (2000).

[14] Humple, D. L. & Porzig, E. L. Riparian landbird monitoring in Golden Gate National Recreation Area and Point Reyes National Seashore: Analysis report through winter 2011–12. Natural Resource Technical Report NPS/SFAN/NRTR—2014/908 (2014)

[15] Sauer, J. R., *et al*. The North American Breeding Bird Survey, Results and Analysis 1966–2015. Version 2.07.2017 USGS Patuxent Wildlife Research Center, Laurel, MD (2017).

[16] Stefani, R. A. The Swainson’s Thrush Survey in the Sierra Nevada Bioregion. Final Report, Univ. of California, Davis (2000).

[17] Linsdale JM (1936). The birds of Nevada. Pacific Coast Avifauna..

[18] Orr, R. T. & Moffitt, J. *Birds of the Lake Tahoe Region*. (California Academy of Science, San Francisco, CA, 1971).

[19] Marshall JT (1988). Birds lost from a giant sequoia forest during fifty years. Condor..

[20] Beedy, E. C. & Granholm, S. L. *Discovering Sierra birds, Western Slope*. (Yosemite Natural History Association and Sequoia Natural History Association, San Francisco, 1985).

[21] Siegel, R. B. & DeSante, D. F. Version 1.0. The Draft Avian Conservation Plan for the Sierra Nevada Bioregion: Conservation Priorities and Strategies for Safeguarding Sierra Bird Populations. Institute for Bird Populations report to California Partners in Flight (1999).

[22] Cormier RL, Humple DL, Gardali T, Seavy NE (2013). Light-level geolocators reveal strong migratory connectivity and within winter movements for a coastal California Swainson’s Thrush population. Auk..

[23] Samuels IA, Gardali T, Humple DL, Geupel GR (2005). Winter site fidelity and body condition of three riparian songbird species following a fire. Western N. Am. Naturalist..

[24] Jennings S, Gardali T, Seavy NE, Geupel GR (2009). Effects of mist netting on reproductive performance of Wrentits and Song Sparrows in central coastal California. Condor..

[25] Rappole JH, Tipton AR (1991). New harness design for attachment of radio transmitters to small passerines. J. of Field Ornithology..

[26] North American Banding Council. North American Bander’s Study Guide, North American Banding Council Publication Committee. http://www.nabanding.net/other-publications/ [accessed 24 June 2019] (2001).

[27] Delmore KE, Fox JW, Irwin DE (2012). Dramatic intraspecific differences in migratory routes, stopover sites and wintering areas, revealed using light-level geolocators. Proc. of the Royal Society of London, Series B..

[28] Lisovski, S., Sumner, M. D. & Wotherspoon, S. J. TwGeos: Basic data processing for light based geolocation archival tags. Github Repository, Retrieved from https://github.com/slisovski/TwGeos (2015).

[29] R Core Team. R: a language and environment for statistical computing, R Foundation for Statistical Computing, Vienna, Austria (2017).

[30] Wotherspoon, S. J., Sumner, D. A. & Lisovski, S. R Package SGAT: solar/satellite geolocation for animal tracking. GitHub Repository, Retreived from https://github.com/SWotherspoon/SGAT (2013).

[31] NatureServe. NatureServe Web Service. Arlington, Virginia. Available at services.natureserve.org. (2012).

[32] Hansen, M. C., *et al*. High-resolution global maps of 21st-century forest cover change. *Science*. ***342***, 850–853 Data (v1.5) available at http://earthenginepartners.appspot.com/science-2013-global-forest. (2013). 10.1126/science.1244693 24233722

[33] Ruegg K (2007). Divergence between Subspecies Groups of Swainson’s Thrush (*Catharus ustulatus ustulatus* and *CU swainsoni*). Ornithol. Monographs..

[34] McElaney, S., Contrasting non-breeding ecology of Swainson’s Thrush (Catharus ustulatus) in Andean forest and shade-grown coffee plantations. Master’s Thesis, Western University (2019).

[35] Kondolf, G. M., Kattlemann, R., Embury, M. & Erman, D. C. Status of Riparian Habitat. *In* Sierra Nevada Ecosystem Project, Final report to Congress, volume II. Assessment and scientific basis for management options. University of California, Centers for Water and Wildland Resources, Davis. 1009–1030 (1996).

[36] Myers N, Tucker R (1987). Deforestation in Central America: Spanish Legacy and North American Consumers. Environ. Review..

[37] Galbraith H, DesRochers DW, Brown S, Reed JM (2014). Predicting vulnerabilities of North American shorebirds to climate change. PloS One..

[38] Toews DP, Taylor SA, Streby HM, Kramer GR, Lovette IJ (2019). Selection on VPS13A linked to migration in a songbird. Proc. of the Nat. Academy of Sci..

[39] Delmore KE, Irwin DE (2014). Hybrid songbirds employ intermediate routes in a migratory divide. Ecol. Letters..

[40] Both C, Visser ME (2001). Adjustment to climate change is constrained by arrival date in a long-distance migrant bird. Nature..

[41] Moussus JP, Clavel J, Jiguet F, Julliard R (2011). Which are the phenologically flexible species? A case study with common passerine birds. Oikos..

[42] Hurlbert AH, Liang Z (2012). Spatiotemporal variation in avian migration phenology: citizen science reveals effects of climate change. PLoS One..

[43] Martin TG (2007). Optimal conservation of migratory species. PLoS ONE..

[44] Tonra CM (2019). Concentration of a widespread breeding population in a few critically important nonbreeding areas: migratory connectivity in the Prothonotary Warbler. Condor..

