# Supplementary material for: Migration tracking reveals geographic variation in the vulnerability of a Nearctic-Neotropical migrant bird
Source: Sci Rep. 2020 Mar 26;10:5483. doi: 10.1038/s41598-020-62132-6 (PMC7099063; doi:10.1038/s41598-020-62132-6)
Supplement: Supplementary file 1 — Supplementary Information. [file 41598_2020_62132_MOESM1_ESM.pdf]

Supplementary Information

Title: Migration tracking reveals geographic variation in the vulnerability of a Nearctic-Neotropical migrant bird

Authors Diana L. Humple[1*], Renée L. Cormier[1], T. Will Richardson[2], Ryan D. Burnett[1], Nathaniel E. Seavy[1,3], Kristen E. Dybala[1], Thomas Gardali[1]

[1] Point Blue Conservation Science, 3820 Cypress Drive # 11, Petaluma CA 94954 USA

[2] Tahoe Institute for Natural Science, 948 Incline Way, Incline Village, NV 89451 USA

[3] Current address: National Audubon Society, 220 Montgomery St, Suite 1000, San Francisco, CA 94104 USA

* corresponding author dhumple@pointblue.org

Supplementary Table S1. Tagging details by location for the Swainson's Thrush 2014-15 deployment effort in central coastal California and two regions within the Cascade-Sierra, combined with data from the 2010 tagging coastal effort as part of an early phase of this study[1].

Region / Locality	Study Site	# Tagged: Light-level, GPS (Female: Male)	Deployment Lat, Lon	Deployment Year
Coastal Region, San Francisco Bay Area, California				
Point Reyes area, coastal Marin County, north of San Francisco Bay	Palomarin Field Station, Point Reyes National Seashore	13[a], 7[b] (14:6)	37.93°, -122.74°	2010[a]<br>2015
	Muddy Hollow, Point Reyes National Seashore	11[a], 9[b] (9:11)	38.05°, -122.87°	2010[a]<br>2015
	Pine Gulch Creek, Bolinas Lagoon Open Space Preserve	11[a], 9[b] (7:13)	37.92°, -122.69°	2010[a]<br>2015
	Redwood Creek, Golden Gate National Recreation Area	0, 5[b] (2:3)	37.87°, -122.58°	2015
coastal San Mateo County, south of San Francisco Bay	TomKat Ranch, San Mateo County	10[c], 0 (5:5)	37.25°, -122.35°	2014
Cascade-Sierra, California				
Tahoe region, northern Sierra Nevada	General Creek, Sugar Pine Point State Park, El Dorado County	2[d],0 (0:2)	39.03°, -120.16°	2014
	Strawberry Creek, El Dorado National Forest, El Dorado County	3[d],0 (1:2)<br>6[d],3[b] (2:7)	38.78°, -120.14°	2014<br>2015
	Ward Canyon, Lake Tahoe Basin Management Unit, Placer County	7[d],0 (1:6)<br>6[d],2[b] (1:7)	39.13°, -120.22°	2014<br>2015
Lassen region, northern Sierra Nevada and southern Cascades, Plumas County	Warner Valley, Lassen National Forest, Cascades	8[d], 10[b] (2:16)	40.42°, -121.34°	2015
	Humbug Valley, Sierra Nevada	3[d],0 (0:3)	40.14°, -121.23°	2015

[a] Mk12-S and Mk10-S geolocators developed by the British Antarctic Survey with 15 mm stalks[1]
[b] PinPoint8 GPS tags developed by Lotek Wireless
[c] PF0C214 light-level tags developed by the British Antarctic Survey with 14 mm stalks
[d] Intigeo-P65B1-14 light-level tags developed by Migrate Technology Ltd with 14 mm stalks

Supplementary Figure S1. Wintering locations of individual Swainson's Thrushes tagged at each of three breeding regions (the coastal San Francisco Bay region, including data from the earlier phase of this study[1]; and Lassen and Tahoe regions within the Cascade-Sierra) in California, with light-level geolocator tag error bars and the original kernel density outlines prior to clipping to species range. Dark gray indicates the published breeding and winter ranges[2]; the authors know of additional breeding sites slightly beyond these boundaries, including where Lassen birds were tagged.

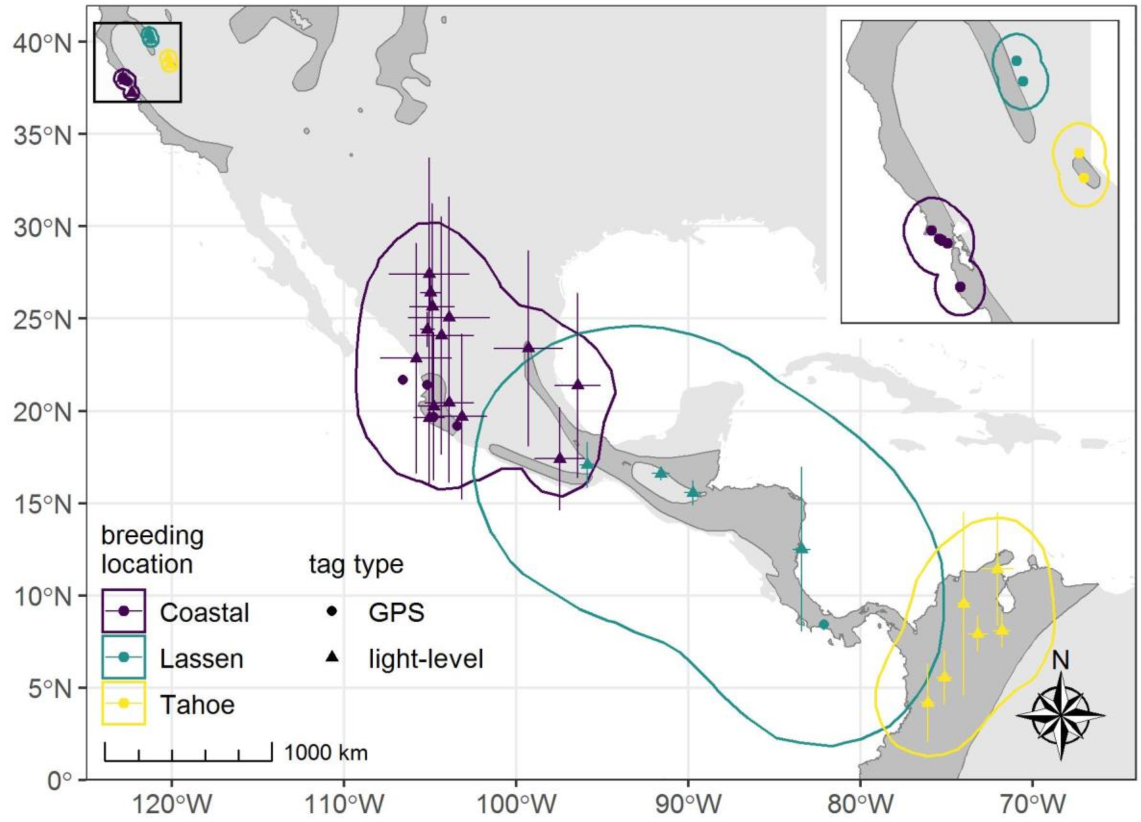

Supplementary Figure S2a-c. Complete individual light-level results for all recovered tags from the 2014-16 phase of the study for the (a) Lassen, (b) Tahoe, and (c) coastal regions. We include the map for one Tahoe bird (tag N918) that we exclude from the overall results, whose wintering areas varied from as far north as Cuba to as far south as Peru depending on minor variations in the analysis method (e.g., changing calibration period dates). See earlier publication for tag data from the previous phase of the study[1], and Supplementary Figure 1 for GPS tag data. Migratory "pathways" are shown on each map in yellow; the more northerly ones are from fall migration, and due to birds migrating around the equinox when latitude cannot be estimated, should not be considered accurate depictions of the migratory routes.

2a. Lassen Region Birds

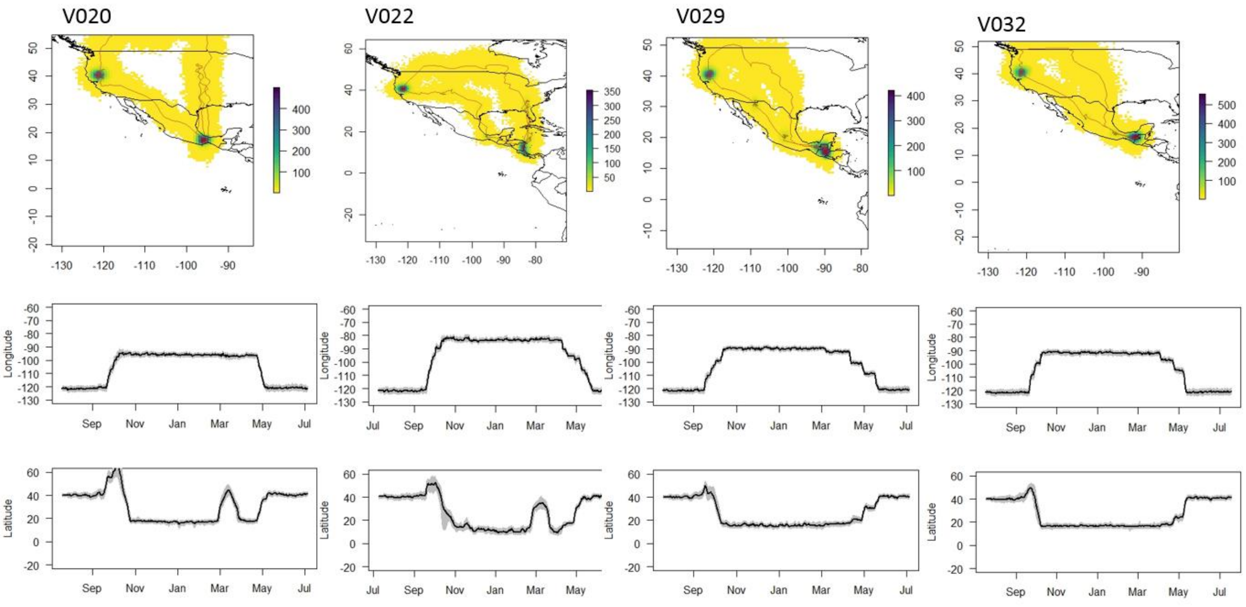

2b. Tahoe Region Birds

N912

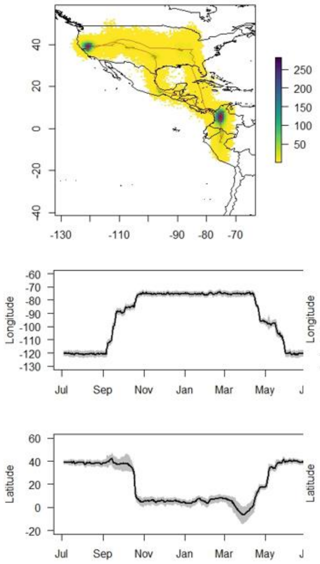

N913

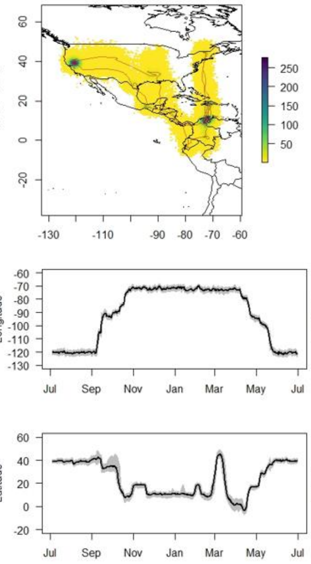

N914

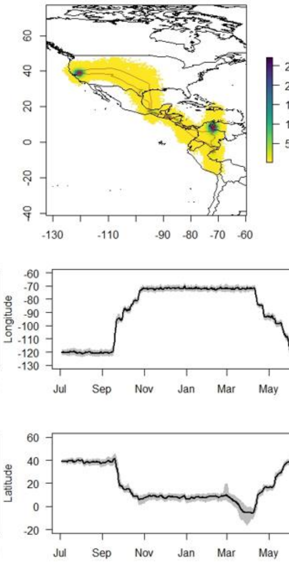

N918

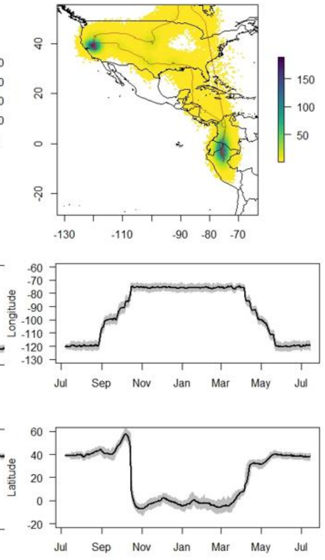

V011

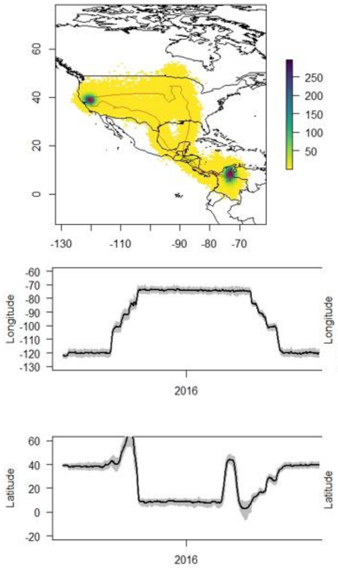

N920

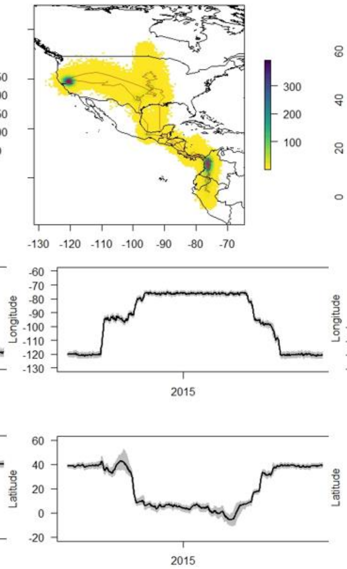

V013

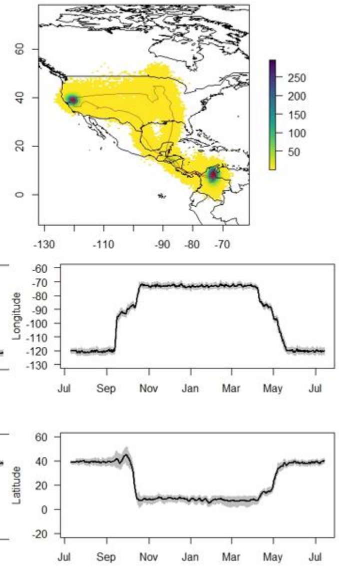

2c. South San Francisco Bay Birds (Coastal Region)

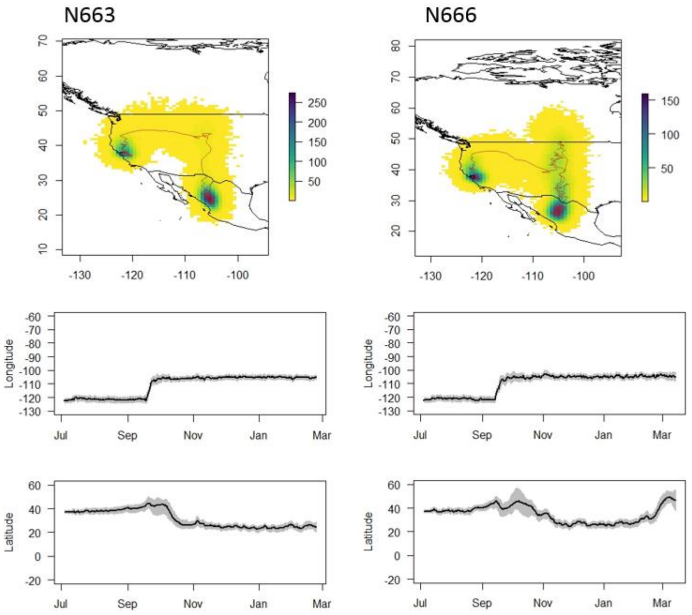

REFERENCES

1. Cormier, R. L., Humple, D. L., Gardali, T. & Seavy, N. E. Light-level geolocators reveal strong migratory connectivity and within winter movements for a coastal California Swainson's Thrush population. *Auk*. 130, 283-290 (2013).

2. NatureServe. NatureServe Web Service. Arlington, Virginia. [Online.] Available at services.natureserve.org (2012).
